# Suprasellar Germinoma Presenting with Slipped Capital Femoral Epiphysis: Case Report

**DOI:** 10.7759/cureus.1954

**Published:** 2017-12-16

**Authors:** Keerthana Sankar, Wade Kyono, Corey Raffel, Theodore Nicolaides

**Affiliations:** 1 Medical School, Wayne State University School of Medicine; 2 Department of Pediatric Oncology, Kapiolani Medical Center for Women and Children; 3 Department of Neurosurgery, University of California - San Francisco; 4 Department of Pediatrics, Department of Neurosurgery, University of California - San Francisco

**Keywords:** scfe, slipped, capital, femoral, epiphysis, pediatric, endocrine, germinoma, suprasellar, tumor

## Abstract

Slipped capital femoral epiphysis (SCFE) is a fracture that results from displacement of the proximal femoral epiphysis from the femoral neck. SCFE can be caused by various endocrinopathies that lead to bone weakening in both adult and pediatric patients. We report a rare case of suprasellar germinoma presenting with SCFE in an 11-year-old female patient. The findings of this case further support the need to consider pituitary lesions as the underlying cause of endocrine deficiences leading to SCFE.

## Introduction

Slipped capital femoral epiphysis (SCFE) is the most common hip disorder in children and occurs in 10.8 cases/100,000 children [1]. SCFE most commonly presents in obese children at an average age of 12.2 with a four-fold higher incidence in Polynesian children and two-fold higher incidence in African American children [1]. Association between various endocrinopathies and SCFE have been found with hypothyroidism being the most common [2]. While it is noted that endocrine disorders are more prevalent in SCFE patients than in the general pediatric population [3], the primary diagnosis can vary. A study of SCFE patients with endocrine disorders shows hypothyroidism, growth hormone deficiency, hypogonadism, or panhypopituitarism as common primary diagnoses [4]. We report an 11-year-old female patient who presented initially with left SCFE and whose medical work-up led to the diagnosis of a suprasellar germinoma. A search of the literature shows that no cases of SCFE associated with suprasellar germinomas have previously been reported.

## Case presentation

An 11-year-old female patient of Samoan (Polynesian) descent presented with left SCFE and acute rheumatic fever. The patient had progressive severe pain in the left hip, difficulty walking, and loss of appetite. The patient did not report skin lesions or symptoms of pharyngitis/respiratory infection. Both parents and siblings were healthy with no reported family history of diabetes, coronary heart disease, asthma, or cancer.

The patient was transferred to a tertiary medical center for hip surgery.  At that time, patient’s review of systems showed polydipsia and polyuria which revealed concern for diabetes insipidus. She had no headaches, emesis, or visual complaints. Further endocrine work-up also revealed adrenal insufficiency, likely growth hormone deficiency, and hypothyroidism. Patient also presented with musculoskeletal abnormalities including hip deformity, arthralgia, and gait problems, all consistent with SCFE. On examination, patient had varus on the right side, 3+ patellar and Achilles Tendon reflexes on the left side, and 3+ right/1+ left suprapatellar reflexes. Gait examination was limited by SCFE but was noted to be non-ataxic and narrow-based. Patient had normal hip abduction and adduction. All other systems reviewed were negative for findings (full skin was not examined due to patient fatigue but was negative for café-au-lait macules at axilla, upper back, and legs). Endocrine findings and other notable lab values are reported in Table 1.

**Table 1:** Patient Lab Values TSH: Thyroid Stimulating Hormone FT4: Free Thyroxine ACTH: Adrenocorticotropic hormone IGF-1: Insulin-like Growth Factor 1

Lab Values	Patient Results	Normal Range
TSH (mIU/L)	2.26	0.28-4.53
FT4 (ng/dL)	0.5 (Low)	0.6-1.3
ACTH (pg/mL)	33	6-55
AM Cortisol (mcg/dL)	3.4 (Low)	6-23
IGF-1 Somatomedin C (ng/mL)	57 (Low)	132-376
Urine Osmolality (mOsm/kg)	47 (Low)	50-1200
Urine Specific Gravity	1.000 (Low)	1.000 – 1.030

**Figure 1:**
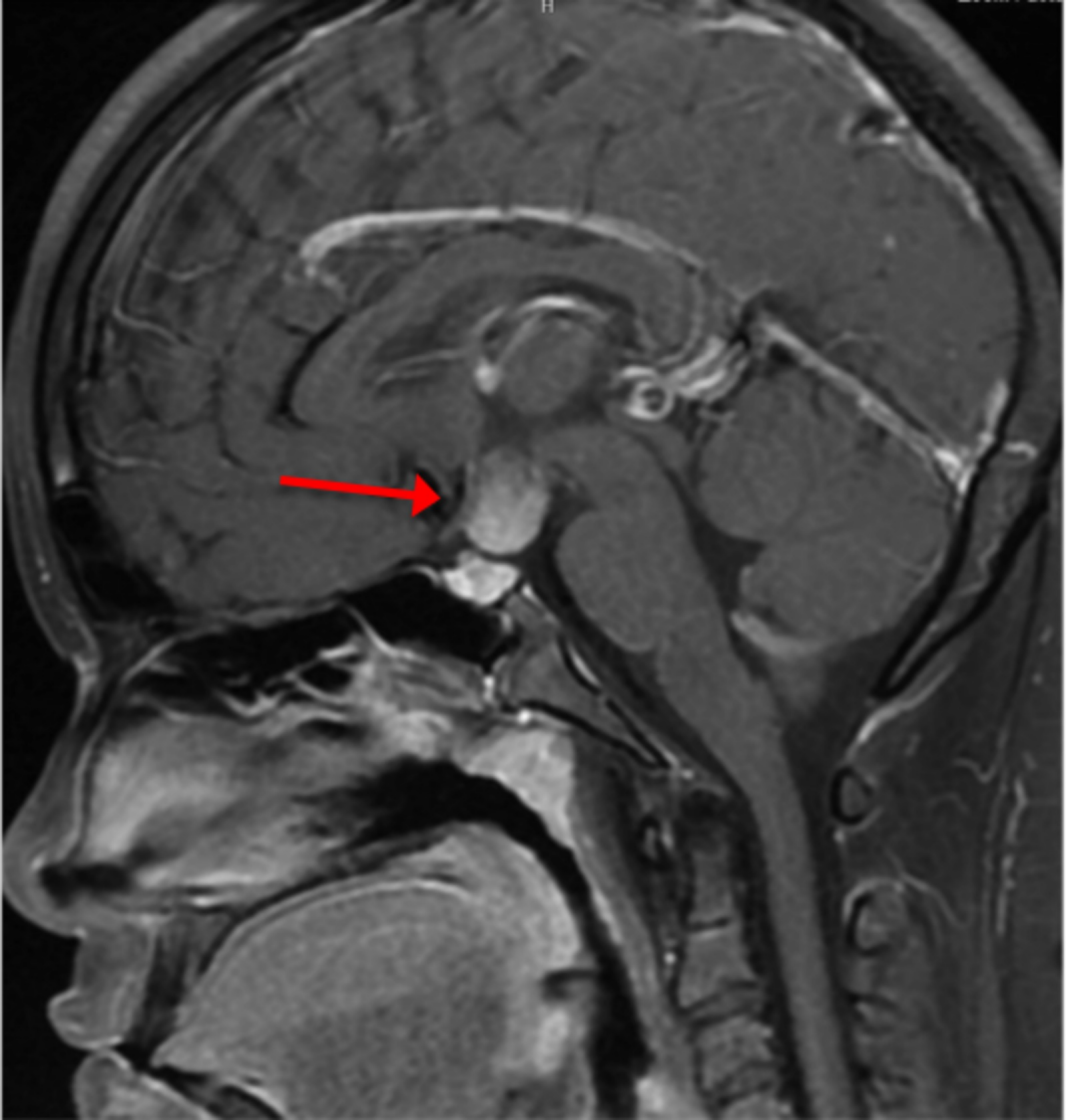
Pre-diagnosis MRI Scan Sagittal T1 post-contrast MRI prior to diagnosis indicates presence of 1.6 x 1.2 x 1.2 cm suprasellar mass.

Magnetic Resonance Imaging (MRI) of the brain following endocrine workup revealed a suprasellar mass of 1.6x1.2x1.2 cm in the upper infundibulum which was deviated to the left and thickened inferiorly (Figure 1­). The pituitary gland was enlarged to 6 mm. Differential diagnosis included diencephalic astrocytoma (including pilocytic and pilomyxoid astrocytoma), craniopharyngioma, germ cell tumor, Langerhans cell histiocytosis, and pinealoma. 

Transsphenoidal biopsy confirmed germinoma, with germinoma cells predominantly occupying the posterior pituitary. Immunohistochemistry was performed on the cells and tested positive for sal-like protein 4 (SALL4), octamer-binding transcription factor 4 (OCT4), and cluster of differentiation 117 (CD117), and negative for pan-cytokeratin, soluble protein-100 (S-100), and cluster of differentiation 1a (CD1a). Serum alpha-fetoprotein (AFP) was 6 ng/mL and serum beta-human chorionic gonadotropin (β-HCG) was reported to be <1 IU/L. Cerebrospinal fluid levels of AFP and β-HCG were <0.5 ng/mL and 2 IU/L, respectively. These findings confirmed a diagnosis of germinoma.  No component of embryonal carcinoma, yolk sac tumor, choriocarcinoma, or teratoma was identified. Patient had no café-au-lait lesions on her skin or other stigmata of neurofibromatosis type 1. Other etiologies to consider included Langerhans cell histiocytosis and lymphocytic hypophysitis. However, there was no other evidence of systemic disease.

The patient underwent the following therapeutic interventions. Her left-sided SCFE was pinned as well as the right side, prophylactically. To treat endocrine abnormalities, the patient was started on desmopressin, hydrocortisone with stress dosing (10 mg/m^2^/day), and levothyroxine. The germinoma was treated with four cycles of carboplatin (600 mg/m^2^/dose x 1 dose) and etoposide (150 mg/m^2^/dose x 3 doses), with each cycle consisting of 21 days. Chemotherapy was followed by 18 gray whole ventricle irradiation with a 12 gray boost to the primary site.

**Figure 2:**
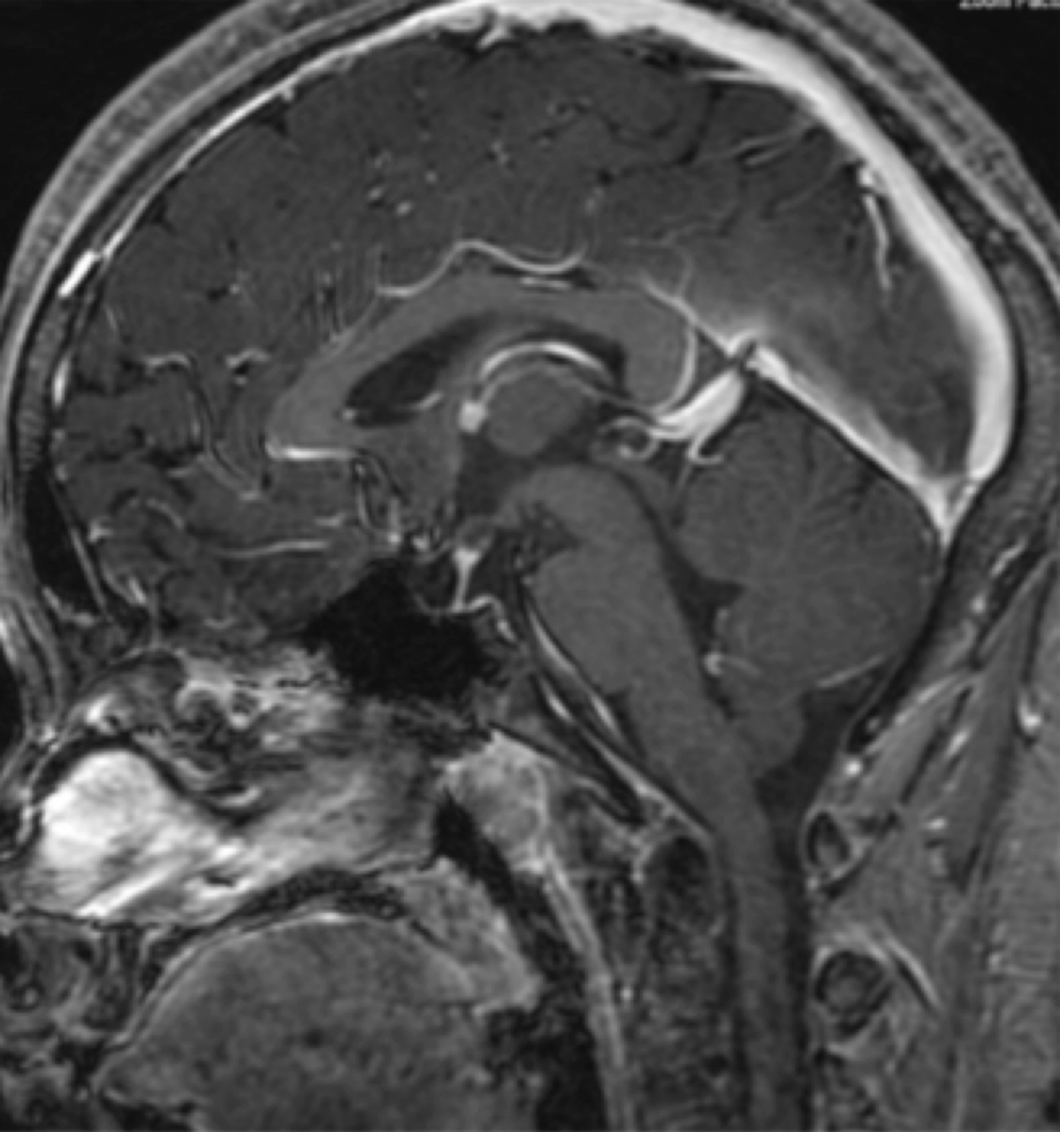
Post-treatment MRI Scan Sagittal T1 post-contrast MRI 32 months after diagnosis indicates no abnormalities.

Following treatment, the patient has done well clinically. MRI of the brain done 32 months from diagnosis showed no suspicious parenchymal, dural, or leptomeningeal lesions (Figure 2). The pituitary gland was decreased in volume and unchanged. The patient has had no further orthopedic problems and remains on hormone supplementation (somatotropin, hydrocortisone, desmopressin, levothyroxine, and estradiol).

## Discussion

SCFE is the most common hip disorder in children between the age of eight and fifteen [5]. Multiple endocrinopathies have previously been reported to cause SCFE, including hypothyroidism, hypogonadism, and panhypopituitarism [4]. Intracranial tumors such as pinealomas, optic gliomas, and craniopharyngiomas have been previously reported in case studies as the underlying cause of endocrine deficiencies leading to SCFE [6-7].

We report a unique case of suprasellar germinoma presenting with SCFE in an 11-year-old patient. Germinomas in children most often present in intracranial and sacrococcygeal regions and are the most common tumors associated with diabetes insipidus, indicating its role in hormone dysregulation [8-9]. While no other case reports of suprasellar germinoma and SCFE are available in the current literature, it is conceivable that the mechanism of action involves lowering FT4 levels, causing bone weakening and leading to SCFE. 

A review of current literature shows that the presence of endocrine disorders in children with SCFE is significantly higher than in the general pediatric population [3]. Various papers have emphasized the need to consider metabolic and endocrine disorders as primary diagnoses for SCFE, especially in patients of noticeable short stature and sexual immaturity [2-3, 10]. We recommend that a pituitary tumor be considered as a possible underlying cause in children presenting with SCFE.

## Conclusions

SCFE is a hip disorder that can occur in both pediatric and adult patients. A significant proportion of SCFE cases are caused by endocrine deficiency. We have identified a suprasellar germinoma as a novel underlying cause of SCFE development.
